# Persistent Calyx Enhances Floral Thermoregulation and Reproductive Success in *Brandisia hancei* Hook. f. (Orobanchaceae)

**DOI:** 10.3390/plants15050795

**Published:** 2026-03-04

**Authors:** Yongquan Ren, Xiangkai Yang, Xin Deng, Ruifeng Sun, Xia Jiang

**Affiliations:** 1 College of Eco-Environmental Engineering, Guizhou Minzu University, Guiyang 550025, China; 2 Guizhou Liping Rocky Desertification Ecosystem Observation and Research Station, Guizhou Academy of Forestry, Guiyang 550005, China

**Keywords:** floral thermoregulation, hance brandisia herb, low-temperature adaptation, persistent calyx, reproductive success, winter-flowering plant

## Abstract

While persistent calyces exhibit considerable functional diversity, this has not been fully substantiated by experiments, especially concerning their thermoregulatory function. This study investigates the thermoregulatory function of persistent calyx in winter-flowering *Brandisia hancei*. Changes in calyx dimensions throughout the flowering-to-fruiting developmental stages were measured. Differences between floral and ambient temperatures were measured when only calyxes were retained. Additionally, differences in floral temperature between calyx-removed treatments and intact controls were also measured. All measurements were taken at three developmental stages: pre-anthesis, anthesis, and post-anthesis. Furthermore, seed production after calyx manipulation was examined at both anthesis and post-anthesis stages. The calyx exhibits continuous size enlargement from flowering to fruiting stages. After either artificial corolla removal or natural corolla abscission, the calyx independently maintains thermoregulatory capacity, sustaining floral temperatures significantly above ambient levels. Consequently, calyx removal resulted in markedly diminished floral temperature at both pre- and post-anthesis stages. In line with the thermoregulation results, progressive removal of the calyx showed a strong negative correlation with seed production. In contrast, removal of only the calyx edge generally maintained seed production at a level comparable to that of the intact control. Collectively, our findings demonstrate that the persistent calyx plays a critical role in elevating reproductive temperature under winter conditions, enhancing reproductive success in *B. hancei* through the maintenance of a favorable thermal conditions for reproduction. This study provides direct evidence that plant reproductive structures can markedly adapt to winter low-temperature stress through such a thermoregulatory mechanism.

## 1. Introduction

The calyx, as the outermost whorl of the flower, primarily functions to protect the developing floral bud. In most plants, it senesces and abscises shortly after flowering; however, certain taxa—including members of the Lamiaceae, Malvaceae, Sapindaceae, Solanaceae, and some clades within Orobanchaceae—retain their calyces after anthesis [1,2]. The persistence of the calyx entails ongoing metabolic costs, suggesting that it confers adaptive benefits sufficient to offset these investments. Previous research has shown that persistent calyces serve multiple ecological roles beyond initial bud protection, including providing physical protection, contributing photosynthetic assimilates and facilitating seed dispersal [3,4,5,6]. Despite these documented functions, experimental evidence remains limited and often taxon-specific. Moreover, further studies are needed to directly link these functions to reproductive output [7].

Cold stress poses a significant threat to plant reproduction by impairing critical processes such as pollination, fertilization, and seed development [8,9,10]. To mitigate this, plants have evolved distinct floral thermoregulation strategies to enhance reproductive fitness, such as passive solar heating via specialized structures that capture and retain solar heat. For instance, bracts (e.g., in *Rheum nobile* Hook. f. & Thomson, *Saussurea velutina* W. W. Sm.) absorb solar radiation, transfer heat inward, and minimize convective loss, thereby creating a stable, warmer microclimate that buffers reproductive tissues against external temperature fluctuations [11,12,13]. The persistent calyx likely functions as an analogous, yet understudied, passive warming structure. In certain species, it forms a hollow or semi-enclosed chamber that can act as a heliocaminiform, regulating floral temperature via a greenhouse effect [12]. Despite the recognized multifunctionality of persistent calyces, their direct contribution to floral thermoregulation and reproductive success under cold conditions remains poorly tested experimentally.

*Brandisia hancei* Hook. f. (Orobanchaceae) is an evergreen shrub that is widely distributed in subtropical China, with its range extending northward to Shaanxi Province [14]. In southwestern China, this plant has been used as a folk medicine by multiple ethnic minorities for treating inflammatory conditions. The isolated compound, Brandioside, exhibits 1.5 to 2 times the antioxidant activity of Trolox, a universal reference standard [15]. This shrub flowers in winter, blooming gradually from late November to early March of the following year. At early developmental stages, the calyx fully encloses the floral bud. The corolla takes about two weeks from emerging from the calyx to being fully open; thereafter, it remains open for approximately one week before withering [16]. Our previous study has demonstrated that its perianth can increase floral temperature under sunny conditions, focusing primarily on the function of corolla [17].

Notably, the calyx—which persists from flowering through fruiting, partly enclosing both the corolla and later the fruit—has a considerably longer lifespan than the corolla. This persistence suggests a more sustained influence across reproductive stages, warranting targeted investigation. However, the conventional approach for functional verification in such studies often relies on complete removal of floral structures, which is compared to intact controls [3,4]. This approach is inherently destructive to the calyx and may compromise reproductive outcomes. More refined experimental treatments are therefore necessary, incorporating not only varying degrees of removal but also a critical additional control: simulating damage by cutting along the calyx margin while preserving its structural integrity and function [10]. The persistent calyx in *B. hancei* is relatively large and thick in texture, making it structurally amenable to such manipulation without compromising its integrity or function. This combination of attributes establishes *B. hancei* as an ideal system for investigating the adaptive significance of persistent calyx under cold conditions.

We address three specific questions in this study: (1) How does calyx size change throughout floral and fruit development? (2) What is the role of the calyx in thermoregulation during key reproductive stages? (3) To what extent does the persistent calyx enhance seed production? By integrating measurements of calyx morphology with manipulative experiments in *B. hancei*, this study provides empirical evidence to elucidate the role of the persistent calyx in reproductive thermoregulation and to assess its impact on plant reproduction in winter.

## 2. Results

### 2.1. Calyx Size

**Figure 1:**
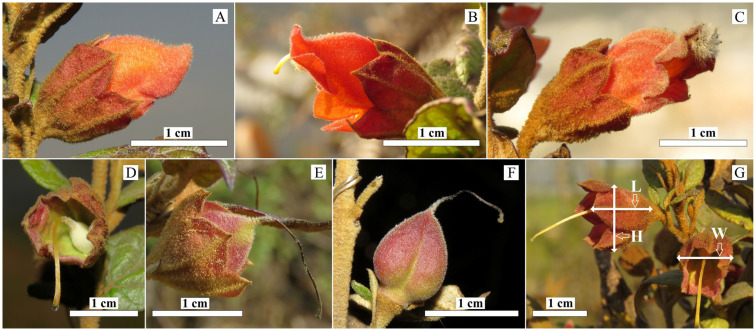
Morphological changes of and measurement method for persistent calyx in *Brandisia hancei*. The calyx partly enclosed the corolla at the stages of pre-anthesis (**A**) and anthesis (**B**); the corolla was about to fall off at the end of anthesis (**C**); the calyx persisted at the stage of post-anthesis, and the ovary developed inside the calyx (**D**); the fruit developed in the persistent calyx at the stage of fructescence (**E**), and fruits also matured without a calyx (**F**). Schematic diagram of calyx measurements (**G**): the length (L), height (H), and width (W) were defined as indicated by the arrows.

**Figure 2:**
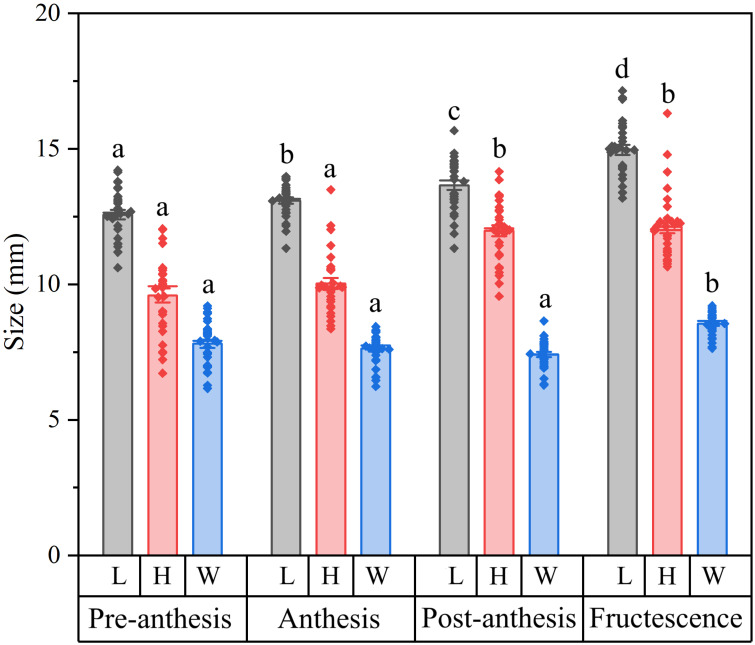
Length (L), height (H) and width (W) of calyx at four developmental stages. Pre-anthesis: flowers were about to open. Anthesis: the corolla was fully open. Post-anthesis: the corolla had abscised. Fructescence: the fruit was developing within the calyx. The values are means ± standard errors; dots represent individual measurement values; *n* = 30. Different letters indicate statistically significant differences (*p* < 0.05).

**Figure 3:**
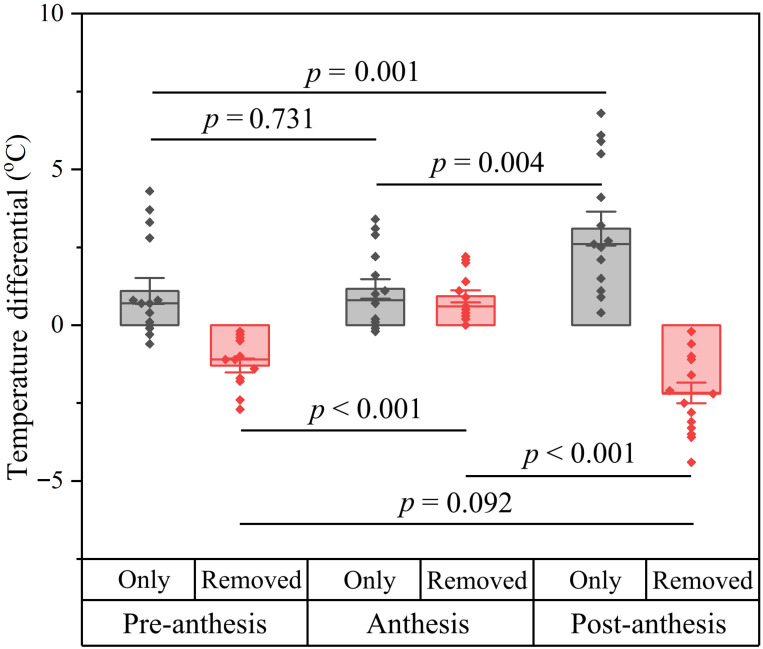
Temperature differentials for flowers at three stages, with calyx only (difference between floral and ambient temperatures) and after the calyx was removed (difference in floral temperatures between calyx-removed flowers and intact controls). Pre-anthesis: flowers were about to open. Anthesis: the corolla was fully open. Post-anthesis: the corolla had abscised. The values are means ± standard errors; dots represent individual measurement values; *n* = 15.

The calyx persisted throughout flowering and fruiting. Based on the morphological changes, the development was classified into four distinct stages: (1) pre-anthesis, when flowers were about to open (Figure 1A); (2) anthesis, when the corolla was fully open (Figure 1B) and abscission was initiated toward the end of this stage (Figure 1C); (3) post-anthesis, when the ovary began to develop within the persistent calyx following corolla abscission (Figure 1D); and (4) fructescence, when fruit developed within the calyx (Figure 1E), although maturation could also proceed without it (Figure 1F).

During the development from flower to fruit, the calyx length (*F_3,116_* = 37.887, *p* < 0.001), height (*F_3,116_* = 33.726, *p* < 0.001) and width (*F_3,116_* = 20.866, *p* < 0.001) maintained continuous growth (Figure 2). During fructescence, the calyx dimensions (length, height, and width) were significantly greater (all *p* < 0.001) than those at both the pre-anthesis and anthesis stages. From post-anthesis to fructescence, the calyx also exhibited a significant increase in both length and width (all *p* < 0.001).

### 2.2. Floral Temperature

Ambient temperatures during the measurements ranged from 12 to 22 °C. The calyx functions independently after both experimental corolla removal at the stages of pre-anthesis and anthesis, and after natural corolla abscission at the post-anthesis stage. The calyx consistently maintained a positive temperature differential, confirming its thermoregulatory function (Figure 3). This effect varied significantly across developmental stages (*F_2,42_* = 7.094, *p* = 0.002). Specifically, while no functional difference was observed between the pre-anthesis and anthesis stages (*p* = 0.731), both showed significantly lower temperatures compared to the post-anthesis stage (pre-anthesis vs. post-anthesis: *p* = 0.001; anthesis vs. post-anthesis: *p* = 0.004).

The impact of calyx removal on floral temperature also exhibited significant stage-dependent variation (*F_2,42_* = 30.114, *p* < 0.001). At both pre- and post-anthesis stages, calyx removal reduced floral temperatures relative to intact controls (Figure 3). In contrast, calyx removal at the anthesis stage increased floral temperature, exhibiting a greater temperature differential compared to both pre- and post-anthesis stages (both *p* < 0.001).

### 2.3. Seed Production

**Figure 4:**
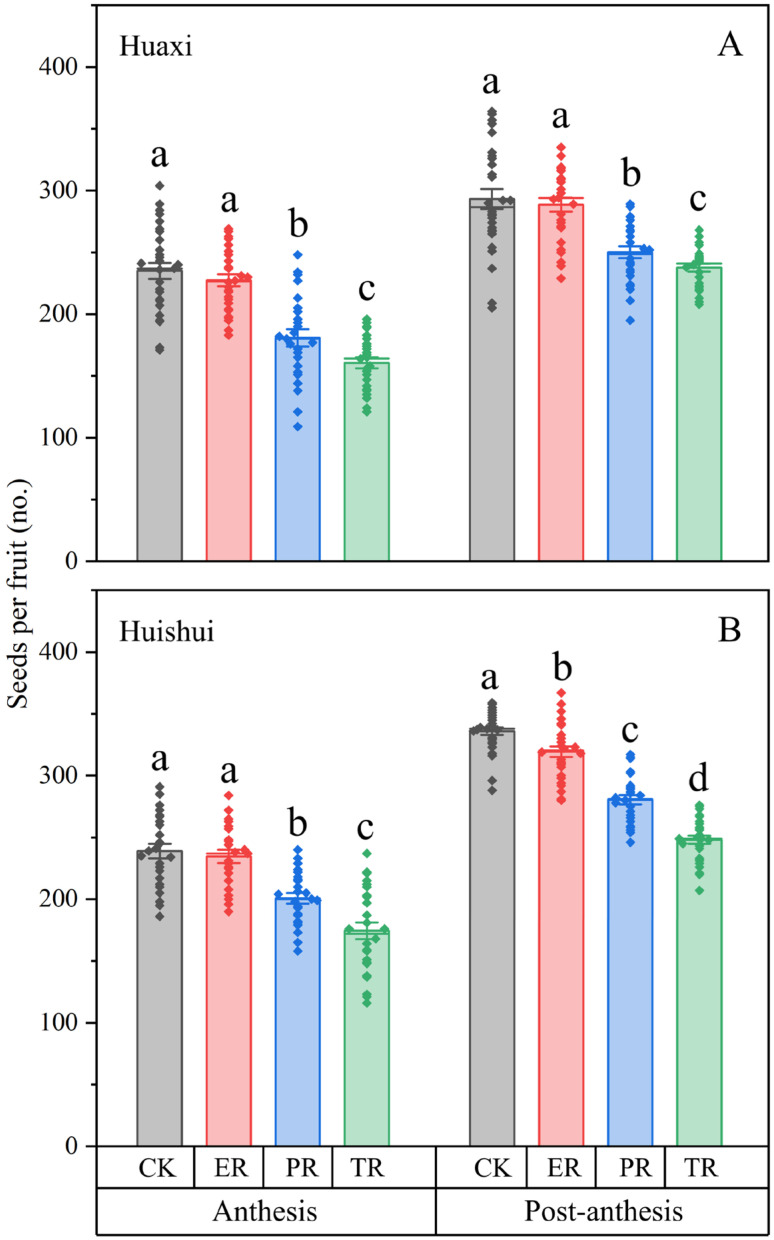
Effects of calyx manipulation at two stages on seed production in populations in Huaxi (**A**) and Huishui (**B**). Anthesis: the corolla was fully open. Post-anthesis: the corolla had abscised. CK, intact control; ER, calyx edge was removed; PR, calyx was partially removed; TR, calyx was completely removed. Data are means ± standard errors; dots represent individual measurement values. Different letters indicate statistically significant differences (*p* < 0.05).

The GLM analysis demonstrated significant effects of both calyx manipulation (Wald *χ*^2^ = 726.957, *df* = 3, *p* < 0.001) and developmental stage (Wald *χ*^2^ = 1030.505, *df* = 1, *p* < 0.001) on seed production in the Huaxi population (Figure 4A). For flowers at both anthesis and post-anthesis stages, pairwise comparisons among treatments (CK, ER, PR and TR) revealed significant differences (*p* < 0.001), with the exception of ER versus CK (*p* = 0.058 at anthesis stage; *p* = 0.315 at post-anthesis stage). Notably, seed production was consistently higher at the post-anthesis stage than the anthesis stage across all treatments, with all stage-wise comparisons showing significant differences (all *p* < 0.001).

Similar results were obtained in Huishui population (Figure 4B), with both treatment (Wald *χ*^2^ = 747.394, *df* = 3, *p* < 0.001) and stage (Wald *χ*^2^ = 1412.153, *df* = 1, *p* < 0.001) showing significant effects on seed production. Pairwise comparisons revealed significant differences among all treatments at both anthesis and post-anthesis stages (*p* < 0.001), with the sole exception of ER versus CK at the anthesis stage (*p* = 0.333). Across all treatments, seed production was significantly higher at the post-anthesis stage compared to the anthesis stage (all *p* < 0.001).

## 3. Discussion

In *B. hancei*, the calyx is already quite considerable at the pre-anthesis stage, partially surrounding the corolla, and continues to grow through flowering and fruiting, ultimately enveloping the developing fruit. Functionally, the calyx functions independently to maintain a warmer floral microenvironment, and calyx removal generally reduces floral temperatures. This floral thermoregulation, achieved through a micro-greenhouse effect, is a passive and ultimately solar-driven process: it converts radiant energy into heat, and consequently, the increase in floral temperature ceases in the absence of sunlight [17]. This is achieved by three key processes: absorption of solar radiation, conductive heat transfer to interior tissues, and reduced convective heat loss [12,13]. Calyx removal produced contrasting thermal effects: it decreased floral temperatures during pre-anthesis but increased them at anthesis. Given the well-established correlation between internal temperature and transmitted light intensity in greenhouses [18,19], the observed rise in floral temperature at the stage of anthesis following calyx removal is most likely attributable to enhanced exposure to sunlight, despite the absence of direct radiation measurements within the flower. For flowers at stage of pre-anthesis, however, calyx removal eliminated thermal insulation. Therefore, even with increased radiation exposure, the corolla alone could not retain heat effectively, underscoring the calyx’s critical role in floral heat retention.

Earlier calyx removal prolonged the negative impact on seed production, which was consistently lower at anthesis than at post-anthesis across populations and treatments. This stage-specific effect likely stems from the removal triggering earlier cold exposure. Cold exposure induces a suite of abnormalities—including tapetum cell degradation, starch particle accumulation in pollen grains, and structural defects in the pollen wall—which collectively undermine pollen vitality and, ultimately, seed set [20,21]. This pattern is further highlighted by an anomaly in the controls: intact flowers at the anthesis stage produced significantly fewer seeds than their post-anthesis counterparts. This discrepancy can be attributed to a natural cold wave in early March, which followed our calyx manipulations in late February 2025. We hypothesize that post-anthesis flowers, with their initiated ovaries, are less susceptible to cold, whereas anthesis-stage flowers suffer direct damage to temperature-sensitive processes like pollen germination and fertilization. This temperature sensitivity is consistent with the documented reproductive impairment during cold events [22]. These findings collectively emphasize the importance of floral warming for reproductive success.

Plants have evolved diverse defenses against insect herbivory, including physical barriers [23,24,25]. Our field observations, however, indicate that the calyx of *B. hancei* is a relatively ineffective physical barrier. We frequently observed herbivorous larvae chewing through the calyx to consume the enclosed fruits. This finding supports studies that suggest that the calyx facilitates larval habitation more than it provides protection [26,27]. Beyond herbivory, the calyx may play a key role in pollination. *B. hancei* has been reported to be pollinated by passerine birds such as *Yuhina nigrimenta* Blyth [28]; our observations confirmed visits by *Pycnonotus xanthorrhous* Anderson, which probes flowers for nectar. When a bird inserts its beak into the corolla tube to feed on nectar, pollen is often deposited on its head. This pollen is then transferred to subsequent flowers that the bird visits, thereby facilitating cross-pollination. Throughout this interaction, the sturdy, campanulate calyx acts as a supportive platform. It cushions the impact from the bird’s head and helps stabilize the floral structure. Thus, although its ability to directly defend against insects appears limited, the calyx’s role in stabilizing flowers during avian pollination constitutes a significant form of protection. Furthermore, the possibility that these structures play a protective role for vulnerable reproductive tissues and early fruit cannot be ruled out [4,6].

Seed production correlated negatively with the extent of calyx removal; however, merely cutting the calyx margin frequently resulted in seed yields comparable to intact controls. This finding argues against the yield reduction being a simple consequence of damage caused by experimental manipulation. Rather, it underscores the functional importance of the persistent calyx itself. The calyx of *B. hancei* exhibits no visible green pigmentation. Furthermore, even in related species with green, photosynthetic calyces, their contribution to final seed yield is often minimal [29,30]. We performed calyx manipulations at both anthesis and post-anthesis stages, consistently finding higher seed production from manipulations at the stage of post-anthesis in both populations. Given the rapid transition from anthesis to post-anthesis in *B. hancei* [16,28], the observed reduction in seed yield following removal cannot be attributed to a loss of photosynthate, even if some photosynthetic potential existed.

Given its limited physical defense against herbivores, the exclusion of manipulative damage as a primary cause of seed loss, and its negligible photosynthetic contribution, the persistent calyx must serve an alternative function. While it does offer some protective benefits to flowers and fruits, the calyx likely plays a more significant role in modulating the thermal microclimate around developing reproductive structures. This hypothesis is strongly supported by the significant negative correlation observed between calyx removal and seed yield. Although humidity may also influence this microenvironment [31,32], temperature regulation appears far more critical for flowers under cold winter conditions—a key thermoregulatory role clearly demonstrated in this study. Warmer temperatures are well-documented to accelerate developmental processes critical for seed maturation [4,33,34]. In *B. hancei*, the hollow calyx absorbs solar radiation, conducts heat inward, and acts as an insulating layer that reduces convective loss, thereby effectively accumulating and retaining heat. Therefore, we propose that a principal function of the persistent calyx is the thermoregulation of developing flowers and fruits, specifically through heat retention.

Considering the potential damage caused by experimental manipulations, this study calls for more refined controls (e.g., edge removal) in future functional studies of plant reproductive biology. Our findings enhance our understanding of the persistent calyx by demonstrating its function in improving the floral microenvironment through thermoregulation under winter conditions. These insights could guide targeted breeding or management strategies to improve reproductive success in crops that possess a persistent calyx or similar, often overlooked structures. Notably, other *Brandisia* species possessing persistent calyces predominantly bloom in summer [14]. This raises intriguing questions about whether their calyces have evolved a divergent thermoregulatory role, such as mitigating heat stress. Investigating the function of persistent calyces in these congeners under summer conditions is therefore warranted. Furthermore, future micrometeorological research could explore the floral humidity maintained within the persistent calyx, as suitable humidity is also crucial for successful fruit and seed development [31,32]. Finally, a complete mechanistic understanding requires a multi-scale approach, extending the mechanistic analysis from the organ level to the cellular and molecular levels [35,36].

## 4. Materials and Methods

### 4.1. Study Sites

Field experiments were conducted in two natural *B. hancei* populations in Guizhou, southwestern China. The first site was in Huaxi (26.3608° N, 106.6614° E; 1105 m a.s.l.), and the second in Huishui (26.1623° N, 106.7634° E; 1245 m a.s.l.). Both sites experience a subtropical monsoon climate with occasional sub-zero winter temperatures [17].

### 4.2. Calyx Size

The length, height and width of the calyx were measured at four developmental stages: pre-anthesis, anthesis, post-anthesis and fructescence. To sample across developmental stages, thirty branches bearing multiple buds from different individual plants in the Huaxi population were labeled. Flowers at various stages were selected from these branches, with only one flower per stage taken from any single branch. Thus, a total of thirty flowers were collected for each developmental stage. Calyx length was measured laterally from the calyx base to sepal tooth apex, and height and width were defined as the maximum vertical and horizontal dimensions of the campanulate structure, respectively (Figure 1G).

### 4.3. Floral Temperature

To investigate the thermoregulatory function of the calyx in *B. hancei*, a series of manipulative experiments were conducted across three developmental stages: pre-anthesis, anthesis and post-anthesis. For flowers with corolla at the pre-anthesis and anthesis stages, we performed two treatments at each stage: (1) preserving the calyx but removing the corolla to clarify the independent role of the calyx, and (2) removing the calyx but preserving the corolla to further assess the thermal contribution of calyx. In the first treatment, temperatures for corolla-removed flowers and ambient air were recorded simultaneously [7,34]. In the second treatment, we conducted paired measurements of calyx-removed flowers and adjacent intact controls at the same developmental stage [17]. For flowers at the post-anthesis stage, defined by natural corolla abscission, only partial calyx removal (removing approximately the upper third while retaining the lower two-thirds) was implemented to evaluate residual thermal effects [17]. Temperature measurements were taken from two adjacent flowers at the post-anthesis stage (one intact control and one manipulated), and the ambient temperature was recorded simultaneously.

All temperatures were measured using multichannel thermocouple thermometers (TA612C, Suzhou TASI Electronics, Suzhou, China) under sunny conditions in the Huishui population. The thermocouple probe consists of a sensing junction and an attached lead wire, and the junction serves as the exact point of temperature detection. To avoid altering the natural state of the flower, the lead wire was first tied to the branch. The probe was then carefully bent so that its sensing junction could be gently inserted and positioned at the basal region inside the flower [7]. For ambient temperature measurements, the sensing junction was shielded with an adjacent leaf. Each treatment included fifteen replicates from distinct individuals, with 10-min continuous recordings at 1-min intervals for each replicate [17]. Due to sunlight availability, 9 replicates were measured on 27 February 2025 (12:00–17:00) and the remaining 6 on 28 February 2025 (14:00–17:30). All treatments were measured concurrently, with measurement time treated as a block (i.e., each measurement time contained a complete set of replicates for all treatments).

### 4.4. Seed Production

Different levels of calyx removal were conducted to assess its role in seed production [10]. To account for potential effects of mechanical stress from the manipulation, an additional treatment involving the removal of only the calyx edges was included. This treatment confirmed that the mechanical stress itself did not significantly affect the results, and the outcomes were comparable to those of the intact controls. Specifically, four manipulation treatments at both anthesis and post-anthesis stages were set up: (1) intact controls (CK), (2) calyx edges were removed to simulate damage without compromising functionality (ER), (3) partial calyx removal as mentioned above (PR), and (4) calyx was completely removed (TR). The calyx removal is carried out using small scissors, without damaging other parts of the flower. For flowers at the anthesis stage, cross-hand pollination (touching the stigma with mature anthers from other individuals) was additionally performed to eliminate the potential effects on pollination caused by calyx manipulation [17]. Thirty flowers from different individuals were used for each treatment, and all four treatments were replicated in the Huaxi and Huishui populations in February 2025. Fruits were then collected for seed counting at the beginning of April. Since some fruits either failed to develop or were damaged by herbivorous larvae, only intact mature fruits were used for seed counting.

### 4.5. Data Analysis

To meet the homogeneity of variance assumption, data for calyx size (length, height and width) were square root transformed (SQRT) and then analyzed by a one-way ANOVA, followed by an LSD post hoc test for multiple comparisons. Statistical comparisons of calyx size were performed across the four developmental stages.

For flowers retaining only the calyx, where the corolla was either removed at the pre-anthesis and anthesis stages or had naturally fallen off at the post-anthesis stage, the temperature differential was calculated as the difference between the floral temperature and the ambient temperature [7,34]. To meet the homogeneity of variance assumption and to handle negative values in the temperature differential, the data were subjected to SQRT (x + 1), after which, a one-way ANOVA and an LSD multiple comparison test were conducted. For flowers subjected to calyx removal, the temperature differential was determined by comparing their temperature with that of adjacent intact controls at the same stage [17]. Due to greater negative temperature differential values in flowers after calyx removal, these data were transformed using SQRT (x + 5). In addition, as the assumption of homogeneity of variance was still violated, a Tamhane’s T2 test was employed for post hoc analysis following the one-way ANOVA. For all treatments, the mean temperature differential was calculated for each replicate based on ten continuous recordings.

To evaluate the effects of calyx manipulation on seed production at two developmental stages, we employed a generalized linear model (GLM) with a Poisson distribution and log-link function to analyze differences in seed production across four calyx-removal treatments: CK, ER, PR and TR. Seed number was treated as the dependent variable, with treatment and developmental stage as fixed factors. All statistical analyses were conducted using IBM SPSS 26.0. (IBM Corp., Armonk, NY, USA).

## 5. Conclusions

The persistent calyx of *B. hancei* continues to grow from flowering to fruiting, initially enclosing part of the corolla and later surrounding the developing fruit. During key reproductive stages, it fulfills a critical thermoregulatory function by forming a specialized microstructure that helps maintain floral temperatures well above ambient levels. Experimental removal of the calyx resulted in a marked decrease in floral temperature across most developmental stages, confirming its essential role in heat retention. Furthermore, the persistent calyx significantly enhances seed production, as evidenced by a strong negative correlation between progressive calyx removal and seed production. Together, these findings provide empirical evidence linking the persistent calyx to floral thermoregulation and reproductive success, deepening our understanding of its functional importance in plant reproduction.

## Data Availability

The data presented in this study can be obtained from the corresponding author upon request.
